# The Heritability of Behaviors Associated With the Host Gut Microbiota

**DOI:** 10.3389/fimmu.2021.658551

**Published:** 2021-05-13

**Authors:** Marcia Manterola, M. Fernanda Palominos, Andrea Calixto

**Affiliations:** ^1^ Programa de Genética Humana, Facultad de Medicina, Universidad de Chile, Santiago, Chile; ^2^ Centro Interdisciplinario de Neurociencia de Valparaíso, Instituto de Neurociencia, Facultad de Ciencias, Universidad de Valparaíso, Valparaiso, Chile; ^3^ Programa de Doctorado en Ciencias, mención Neurociencia, Facultad de Ciencias, Universidad de Valparaíso, Valparaiso, Chile

**Keywords:** transgenerational inheritance, germ cells, small RNAs, host-bacteria interactions, microbiota, behaviors

## Abstract

What defines whether the interaction between environment and organism creates a genetic memory able to be transferred to subsequent generations? Bacteria and the products of their metabolism are the most ubiquitous biotic environments to which every living organism is exposed. Both microbiota and host establish a framework where environmental and genetic factors are integrated to produce adaptive life traits, some of which can be inherited. Thus, the interplay between host and microbe is a powerful model to study how phenotypic plasticity is inherited. Communication between host and microbe can occur through diverse molecules such as small RNAs (sRNAs) and the RNA interference machinery, which have emerged as mediators and carriers of heritable environmentally induced responses. Notwithstanding, it is still unclear how the organism integrates sRNA signaling between different tissues to orchestrate a systemic bacterially induced response that can be inherited. Here we discuss current evidence of heritability produced by the intestinal microbiota from several species. Neurons and gut are the sensing systems involved in transmitting changes through transcriptional and post-transcriptional modifications to the gonads. Germ cells express inflammatory receptors, and their development and function are regulated by host and bacterial metabolites and sRNAs thus suggesting that the dynamic interplay between host and microbe underlies the host’s capacity to transmit heritable behaviors. We discuss how the host detects changes in the microbiota that can modulate germ cells genomic functions. We also explore the nature of the interactions that leave permanent or long-term memory in the host and propose mechanisms by which the microbiota can regulate the development and epigenetic reprogramming of germ cells, thus influencing the inheritance of the host. We highlight the vast contribution of the bacterivore nematode *C. elegans* and its commensal and pathogenic bacteria to the understanding on how behavioral adaptations can be inter and transgenerational inherited.

## Introduction

Microbes present in the environment are the most ubiquitous stimuli to which all organisms are exposed. Microbial ancestors likely shaped the evolution of eukaryotes, establishing mutual adaptation mechanisms for long-term survival and benefit (1). Microorganism and multicellular hosts communicate by a plethora of mechanisms that depend on the nature of their relationship, whether it is of commensalism, mutualism, or pathogenic (2). The outcome of a microbe-host relationship depends on their mutual interaction and cannot be established *a priori* based on the separate parts alone (3).

The recognition of the immediate environment is a key step in the establishment of short and long-term adaptive responses by all organisms. The duration, intensity, and nature of environmental stimuli are the parameters that influence the execution of behavior and its heritability. Brief homeostatic inputs are dealt with as part of the physiological response and likely do not leave traces in organisms’ genetic and cellular memory. Conversely, exposure to persistent threats such as viral or bacterial pathogens generate heritable responses to infection in future generations (4–8).

## Sensing the Biotic Environment and the Creation of a Chemical Memory

The bacterivore nematode *Caenorhabditis elegans* is an exceptional model organism to study host microbiota relationships, and importantly for this review, the interactions that create a multigenerational memory. Nematodes show preference over their bacterial food, display olfactory associative learning (9), and develop transgenerationally inherited strategies to survive pathogenesis (5–8).

Microbes contribute essential metabolites and produce toxins that generate disease. Therefore, it becomes essential for the animal to discriminate between pathogenic *versus* non-pathogenic chemical environments to display appropriate behaviors. Specific olfactory neurons sense soluble and volatile chemicals from the biotic environment (10, 11) to mediate attraction or aversion to pathogenic and commensal bacteria (9, 12–14). Interestingly, a number of potentially pathogenic bacteria are attractive at first since they also produce attractive odorants (15) or contain metabolites of high nutritional value such as vitamin B12 (16). This poses a hard choice for the animal, having to prioritize escaping damage over the potential benefits of good nutrition such as the acceleration of development. Thus, the correct integration of different sensory neuronal circuits allows for the command of behaviors that likely prioritize survival. This learned behaviors are maximally effective if they can be transmitted to the following generations.

*C. elegans* avoids pathogens by activating different G protein-coupled receptors (GPCR) in specialized chemosensory neurons that detect secreted bacterial molecules and orchestrate adaptive behaviors like pathogen avoidance (9, 13, 17–19). Additionally, sensory neurons are able not only to coordinate behavior but also to orchestrate immune responses: The activation of the same GPCR, OCTR-1, in the chemo and thermosensory ASI neuron triggered avoidance, but in the ASH polymodal nociceptor caused a cascade that activates neuropeptidergic immune pathways (20). Sensory neurons thus play a key role in many diverse and complex processes related with pathogen responses, from avoidance and immunity to learning and memory formation.

The gastro-intestinal tract is home to microbes of an immense variety of genus (21, 22) and enteric sensory neurons located next to the intestine (23) can detect changes in the composition of microbe’s metabolic byproducts (24–26). Diverse host’s physiological responses have shown to be modulated by microbial metabolites, like short-chain fatty acids, polysaccharides, bile acids, and others (27), in a bidirectional communication process between host and microbe that is evolutionary conserved across animals (28). Chemical information about the intestinal luminal environment is transmitted to the CNS through the vagus nerve in mammals (29), and through the recurrent nerve in insects, like *Drosophila* (30). In *C. elegans*, sensory neurons detect microbial metabolites such as bacterial autoinducers [homoserine lactones (12), (S)-3-hydroxytridecan-4-one (15), and other virulence factors like phenazine and pyochelin (19). to coordinate behaviors like avoidance (12, 13, 19) and attraction (15). However, it is still unknown how the worm senses intestinal bacterial metabolites. Mammalian intestinal cells respond to bacterial short-chain fatty such as acetate and butyrate through the activation of GPCRs (31) thus suggesting that both neurons and intestinal cells may detect chemical changes in the intestinal lumen. But, how might environmental experiences, decoded by sensory neurons distant from the germline, reprogram the gamete’s genetic memory?

In nematodes, the AWC and AWB chemosensory neurons coordinate sensing of pathogens and aversive olfactory learning (17). Recently, these sensory neurons have also been implicated in promoting intestinal p38 MAPK immune responses to pathogens (32). From the intestine, environmental information can be transgenerationally transmitted to the germ cells through inter-tissue regulation of the expression of ASH-2 and RBR-2 histone-modifiers (33). Taken together these raises the possibility that the transmission process involves communication between the brain, the gut and the germline. To add to this idea, the ASI ciliated chemosensory neuron involved in dauer formation (34, 35) and bacterial metabolite detection (19), controls transgenerational pathogen avoidance (6) and germline development (36, 37). These sensory neurons control germline development by coupling proliferation/differentiation (37) and apoptosis of the germline stem cell pool, with sensing of the environment’s quality by the activation of the DAF-7/TGF-β pathway in parental neurons and DAF-3/DAF-12 in the progeny (36). This suggests that neurons that are continually sensing and detecting changes in the external and internal environment may directly regulate such a delicate process like reproduction. However, how neurons, intestinal, and germ cells coordinate the information extracted from the environment to transmit a long-term behavioral memory associated with changes in the repertoire of local microbe byproducts remain mostly unknown.

The length of parental exposure to pathogens modulates the behavior of future generations when exposed to those same stimuli. For example, the progeny of nematodes exposed to highly virulent pathogens for short periods of time are attracted to the bacteria, while progeny of parents exposed for more extended periods of time inherit pathogenic avoidance, intergenerationally (8). Is it known that the molecular effectors mediating the inheritance of this behavior are dependent on the endo-siRNAs pathway; however, it is not known yet the neuronal circuits involved in its coordination. In a related paradigm, the progeny of nematodes exposed for two generations to mildly virulent pathogens like *P. aeruginosa* PAO1 or *Salmonella enterica* (serovar Typhimurium) enter diapause forming a stress-resistant larva called dauer that does not feed. This response is not observed in the first generation, which implies that the molecular processes that underlie dauer formation under pathogenesis need to build up to a threshold. This depends on the accumulation of molecular damage signals that turn on defensive pathways like FOXO/DAF-16 (5, 38) and RNAi-dependent molecules (5, 7). Interestingly, a change in the pathogen’s virulence or the animal immune status dramatically affects diapause entry (5). This shows that the process of inherited memory formation depends on the duration of the threat but also to the pathogen’s virulence determinants. We propose that the inheritance of a pathogenic memory requires sensory inputs (e.g., olfactory cues and bacterially-derived metabolites), to be integrated with internal signaling factors. These factors include host immunity molecules, and the products of the local host-microbe interaction. Importantly, the memory of pathogenic encounters can be inherited by subsequent generations *via* mechanisms that involve RNA interference pathways (5, 6, 8). This supported the idea that these survival strategies against pathogens maintained for multiple generations in absence of the threat are inherited through mechanisms based on RNA molecules.

## Small RNAs Mediate Transgenerational Inheritance Triggered by Environmental Changes

sRNAs can effectively bridge soma and germline throughout generations. sRNAs are mediators of heritable epigenetic changes in the offspring (39) and are able to sustain intergenerational (40) and transgenerational (41) transmission. Among the heritable sRNAs are short-interference RNAs (siRNAs), microRNA (miRNA), PIWI-interacting RNA (piRNA), RNAs-derived from transfer RNAs (tsRNAs), ribosomal RNAs (rRNA), and circular RNA (circRNA). In germ cells, these sRNAs are key transcriptional and post-translational regulators and bind to Argonaute proteins, and Piwi-interacting RNAs (42, 43). In addition to mRNA silencing, RNA mediated alterations occur at chromatin level (43) and are known as RNA-triggered chromatin modifications. Endogenous siRNAs direct chromatin remodeling by inducing the methylation of specific genomic regions and promoting heterochromatin formation (44). In *C. elegans* and *Drosophila*, siRNAs trigger RNA interference (RNAi) pathways in germ cells that are amplified in the offspring for several generations (39, 45–47). Amplification of siRNAs is also essential for targeting specific genomic loci and inducing histone modifications, creating a footprint that is transgenerationally maintained for at least two generations (43, 48).

piRNAs are sRNAs that are highly expressed in germ cells and are involved in the maintenance of the genome and the initiation of multigenerational epigenetic inheritance. piRNAs expression have been also identified in somatic tissues such as follicle cells, hippocampus, kidney and liver (49) as well as in cancer cells (50, 51). piRNA induce heritable epigenetic modifications to silence specific loci in the genome (52–54) and mediate heritable responses to heat stress, toxicants, high-fat diet (54–59) and avoidance to pathogenic bacteria (6). miRNAs are essential regulators of female and male gametogenesis (60, 61), and embryonic development (62). Alterations in sperm miRNA content due to, for example, stress and metabolic changes, are intergenerationally inherited (63–65). In *C. elegans* miRNAs are highly sensitive to pathogenic exposure and mediate the initiation of transgenerational behaviors like pathogen induced-diapause (7). tsRNAs are enriched in male germ cells and known to inhibit translation, regulate transcription and chromatin modifications (66). tsRNAs can be methylated and have shown to mediate intergenerational transmission of metabolic disorders (67). circRNAs regulate transcription and splicing, modulate translation and post-translational modifications, and act as microRNA (miRNA) sponges (68, 69). circRNAs have been found in seminal plasma, suggesting that they can also mediate a soma-germ cell communication pathway (70). All the above suggests that small RNAs are sensible communicators of environmental variations such as changes in microbiota content. But, can the gut microbiota itself produce sRNAs that reach host tissues beyond the intestine? Or do they produce intestinal effects that propagate systemically? If they can reach the host tissues, do they work as initiators, executers or are they *the memory* required for behavioral outputs?

Gut microbiota influences siRNA expression in the intestine and brain, affecting host transcriptional reprogramming and raising the possibility that microbe-derived RNAs or by-products communicate with somatic cells (71–79). In mice, several reports have showed that microbiota influences transcription of miRNAs in the amygdala and prefrontal cortex (73). Among these miRNAs, miR-183-5p and miR-182-5p (73) are of special interest as they are involved in stress- and fear-related responses (80, 81). Further to this, stress was previously shown to produce the accumulation of miRNA membrane vesicles in the epididymis and testes, thus transmitting an intergenerational response to stress in the offspring (82).

In *C. elegans*, neuron-specific synthesis of sRNAs regulates the amplification of endogenous siRNAs in germ cells, thus changing germline gene expression for multiple generations (83, 84). This mechanism opens the possibility that an olfactory stimulus elicited by bacteria changes the repertoire of endogenous neuronal sRNAs, which can later promote epigenetic changes in germ cells that will affect the offspring’s behavior and fitness. To date, two sRNAs from bacteria have been identified that influence inherited behaviors in *C. elegans*. P11 from *P. aeruginosa* PA14 influences avoidance behavior (85), while the quorum sensing RNA RsmY from *P. aeruginosa* PAO1 is required for diapause formation under pathogenesis (86). How sRNAs from bacteria might be reaching the host soma and germline is discussed later.

## Transport of Interspecies sRNAs Between Host Tissues

Can bacterial molecules such as RNAs use the host transport machinery to reach host tissues and eventually the germline? Much needs to be learned about the import of bacterial metabolites into host cells, and their selectivity. However, several mechanisms have been described that can accommodate such a travel.

There are dedicated mechanisms to export and uptake RNA molecules from other cells and the extracellular space. In *C. elegans*, selective receptors and RNA transporters participate in the systemic movement of RNA molecules (SID proteins). SID-1 is a conserved channel gated by double stranded RNA (dsRNA), ref (87), which allows the passage of dsRNA between *C. elegans* cells and also in heterologous systems (88). Its human homolog - SIDT1- is known to mediate bidirectional, sequence-specific and target-specific small RNA transfer between human cells (89). Other SIDs like SID-3, a conserved tyrosine kinase is required for import of dsRNA in worms (90). SID-5 is an endosome-associated protein (91) with a role in embryonic parental RNAi in *C. elegans* (92). A relevant transporter in bacteria-worm communication is SID-2, which localizes to the intestinal lumen and potentially serves as a gate between host enterocytes and intestinal microbes. This protein is required for the import of ingested dsRNA, through an endocytosis-mediated and energy dependent mechanism (93). SID-2 shares functional similarities with Toll-like receptor 3, which in humans localizes to endosomes and recognizes dsRNA and sRNA (94). Defensive mechanisms such as pathogen-induced diapause formation (PIDF) require intact SID-dependent pathways for dsRNA import (5), opening up the possibility that the bacterial sRNA delivery system into the host uses a SID-dependent entry. It is however unknown how are bacterial RNAs found in the intestine. Are they secreted by selective bacterial exocytic mechanisms? Alternatively, can they be contained in membrane vesicles or exposed as cytosolic contents after the explosive stress-induced lysis of bacteria?

Bacterial Outer Membrane Vesicles (OMVs) are able to carry RNA among other cargoes (95–99). Increasing research has revealed the importance of OMVs RNA-cargo in bacteria-host interactions (99–101). Other types of bacterial vesicles are membrane vesicles produced by explosive lysis of both Gram-positive and Gram-negative bacteria (102, 103). This, a seemingly stochastic process, generates MV containing specific cargoes. This raises the possibility that bacterial vesicles use the host endocytic pathway to deliver cargoes to specific tissues (104). To induce intergenerational silencing in *C. elegans*, injected dsRNA can be transported to the oocyte from the worms’ body cavity within intracellular vesicles that depend on the LDL receptor superfamily homolog RME-2 (92). The existing molecular machinery regulating the inheritance of dsRNA through RME-2 (92, 105, 106) and the capacity of the bacteria to generate OMVs with RNA cargoes suggests that bacterial RNAs present in the intestinal lumen may enter the host cells by two separate but synergic mechanisms: endocytosis-dependent and endocytosis-independent pathways (like SIDs RNA transporters).

It has been shown that RNA can be transported from brain to the germline, and from there inherited to the embryos (107). This demonstrates that sRNAs are central players in soma to germline communication pathways. Within this interaction pathways, sRNAs can be transported through exosomal and non-exosomal transport *via* blood and follicular fluid to oocytes (108, 109), and *via* seminal and epididymal luminal fluid to sperms, breast milk, saliva, and cerebrospinal fluid (74, 110). sRNAs can also be loaded onto sperms by extracellular vesicles present in the outer membrane of epididymal cells (111, 112). Several of them are also transferred through RNA transporters. That is, in *C. elegans*, the RNA transporter SID-1 actively participates in transporting extracellular dsRNA into oocytes and in the inheritance of siRNAs (92). Therefore, RNA delivery is an active mechanism that allows germ cells to acquire information from somatic cells. We expect that future work will shed light onto questions regarding the origin, transport and systemic internalization of bacterial sRNA in the host.

## Epigenetic Inheritance of Environmental Experiences: Can Bacteria Modify the Germline?

Any new environmental experience that affects individuals has the potential to modify the epigenome. When modifications in the chromatin state and in the RNA content occur in germ cells, the epigenetic memory could be transmitted across subsequent generations. A change in the epigenetic profile of germ cells is therefore a mandatory step to inheritance of environmental adaptations (111). Direct evidence shows that DNA methylation, histone modifications, histone variants, and non-coding RNAs transferred by germ cells can promote phenotypical adaptations to the offspring for several generations in the absence of the stimulus that initiated the response (111, 113, 114). But, can bacterial molecules cause such changes? Until our knowledge, there is no evidence showing a direct effect of gut bacteria over the germ cells. However, by considering that it is very hard to demonstrate this experimentally, we cannot exclude this possibility.

Although the mechanisms that link gut microbiota and the epigenetic modifications in germ cells are largely unknown, the influence of bacteria in the host epigenome has been unequivocally demonstrated (115). Microbial signals including ncRNAs, metabolites and inflammatory molecules interact with histone writers such as DNA methyltransferases (DNMTs) and histone deacetylases (HDAC) or regulate the availability of writer’s substrates such as SAM, the primary methyl donor for DNMT (115). Interestingly, at least histone modifications and sRNAs have been shown to be related to a potential gut bacteria-neural- germ cell cross talk, highlighting the importance of gut microbiota in the inheritance of phenotypical traits (116, 117).

Recently, a link between gut microbiome, the epigenome of germ cells and the offspring´s behavior has been suggested in *C. elegans*. Intestinal distention produced by bacteria induces acetylation in histone 4 in germ cells, particularly H4K8ac (116). This mark could be sufficient to inherit pathogen avoidance in the offspring, underlying the still unknown mechanism of crosstalk between sensorial neurons, brain, gut and germline to control transgenerational phenotypes. Furthermore, it raises the possibility that mechanical changes in the intestinal epithelium may also drive the production of host-derived metabolites and sRNAs, thus allowing inter-tissue and intergenerational transmission of information.

Histone Post-Translational Modifications (PTM), as well as histone-writers and reader proteins are critical regulators of the gene expression programs in both the germline and the zygote. From *C. elegans* to the mouse, several PTMs in sperms and oocytes including mono and di-methylations of histone H3 at lysine 4 (H3K4me1/me2) and trimethylation of histone 3 at lysine 9 (H3K9me3), are retained at genomic regulatory elements (118), imprinted regions and miRNA clusters (119, 120). Disruptions in the histone retention regions in sperms produced by environmental stimuli can transgenerationally persist up to the third generation (121–123). Although the evidence of bacterial-induced acetylation in germline epigenome needs to be further supported, the possibility that gut microbiota influence PTM in germ line provides a hint in how bacteria produce environmental changes that are sensed and inherited to the progeny. Whether gut bacteria directly affect PTMs in germ cells or it is a secondary effect after a mechanical or chemical stimulus needs to be further clarified.

## Bacterial Metabolites and Their Effect on Behavior

Bacteria are true metabolite factories, producing a large number of neurotransmitters, vitamins among other essential nutrients. It is clear that microbial products affect life history traits of the individuals carrying them. However, can bacterial metabolites such as amino acids, fatty acids and other, influence the progenies inter or transgenerationally? Naturally, whether it is directly or indirectly, the transformation leading to inheritance is stored in the germline. While the contribution of sRNAs in inheritance has been widely documented, transmission of life history traits by metabolites is less clear. Bacterial metabolites orchestrate epigenetic pathways: Inositol phosphate (IP3) influences histone acetylation in the intestine by regulating histone deacetylase 3 (HDAC3) activity (124). Other bacterial metabolites, like folate, had shown to regulate the proliferation of the germline and the fertility of *C. elegans* (125). Although the presence of bacterial genetic material or metabolites has not been directly observed in tissues other than the intestine, it is formally possible that they can reach the germline. Bacterial metabolites and sRNAs stimulate the soma-to germ line communication axis as well as immune responses that could modulate the epigenetic programing and even immune priming in germ cells (111, 126–130). The interactions between histone modifications, sRNAs and transmissible chromatin domains provide a framework in which metabolites may produce transgenerational epigenetic effects in gametes.

The absence of intestinal microbiota is well known to cause impairment in the development of the brain (131) and the enteric nervous system (132, 133). Bacteria can synthesize neurotransmitters like GABA, dopamine, serotonin, melatonin, histamine and acetylcholine (29, 134, 135), which posses’ receptors in most animals. Germ-free mice showed reduced enteric contractibility (132) thus suggesting that maybe the lack of a bacterially produced neurotransmitter underlies the observed developmental abnormalities. It was in the late 80’s that Minuk (136) suggested for first time that human mood can be modulated by bacterial metabolites: the loss of consciousness seen in patient with total septis or liver disease can be related with an increase in GABA production from pathogenic bacteria colonizing the bowel (136). Recently this idea has been retaken and defined as ‘psychobiotics’, probiotics with ‘mind-altering’ capacities (137). This new concept leads to think that bacterial metabolites can make their effect directly onto enteric neurons or intestinal epithelial cells to induce the release of a range of host modulatory molecules that can regulates diverse processes like immunity and behavior. Likewise, the intestinal bacterial metabolome of the honeybee specifically regulates the bee’s nursing behavior and the host’ circulating metabolites in the hemolymph/blood (138). How changes in the physiology provoked by altering the bacterial metabolome profile are integrated and transmitted to the progeny of complex hosts, like mammals, are yet remain to be elucidated.

## Cytokine Signaling as a Mechanism to Influence Transgenerational Inheritance in Germ Cells

The intestinal biota and its metabolites not only modulate the brain but also the immune system (27, 139). This interaction is a dynamic process essential in maintaining homeostasis, which involves the recognition and tolerance against commensal microbiota, or the battle against pathogens. However, it is still unknown how changes in the immune system, produced by variations in the intestinal microbiota composition, may impact transgenerational memory formation. Research in *C. elegans* has recently shed light on the intergenerational maintenance of microbe-induced immune transcriptional changes (6, 7). Thus, and similarly than the inheritance of the avoidance behavior, parental encounters with bacteria can lead to primed immune genes in germ cells that allow robust transcriptional responses against future infections in the offspring.

Cytokines are circulating factors produced by immune cells creating a communication pathway between the immune system and the brain, modifying brain function and behavior (140, 141). Furthermore, cytokines modulate mammalian germ cell development and differentiation, in both female and male gonad function. In females, macrophage secretion of TNF-α and interferon-γ (IFN) stimulate the development and degeneration of the corpus luteum and, in so doing; regulate ovarian function (142). In the testis, Sertoli and Leydig cells produce numerous cytokines (like TNFα, IL1, IL6, and IL18), which act as a paracrine signal that regulates germ cell development and function (143–147). In macrophages, IFN induces chromatin remodeling and inherited transcriptional immune memory (148), and modifications of histone marks like H4ac, H3K9ac, and H3K4me3 at IFN-activated promoters (149). Although these data support the idea that regulation of the host’s immune system controls some aspects of transgenerational inheritance, how microbial-induced cytokine production in the gut may modulate epigenetic changes in germ cells is still mostly unexplored. Future research will be invaluable in resolving how microbial-induced cytokine production influences germ cells and its impact on the host inheritance capacity.

## Discussion

**Figure 1:**
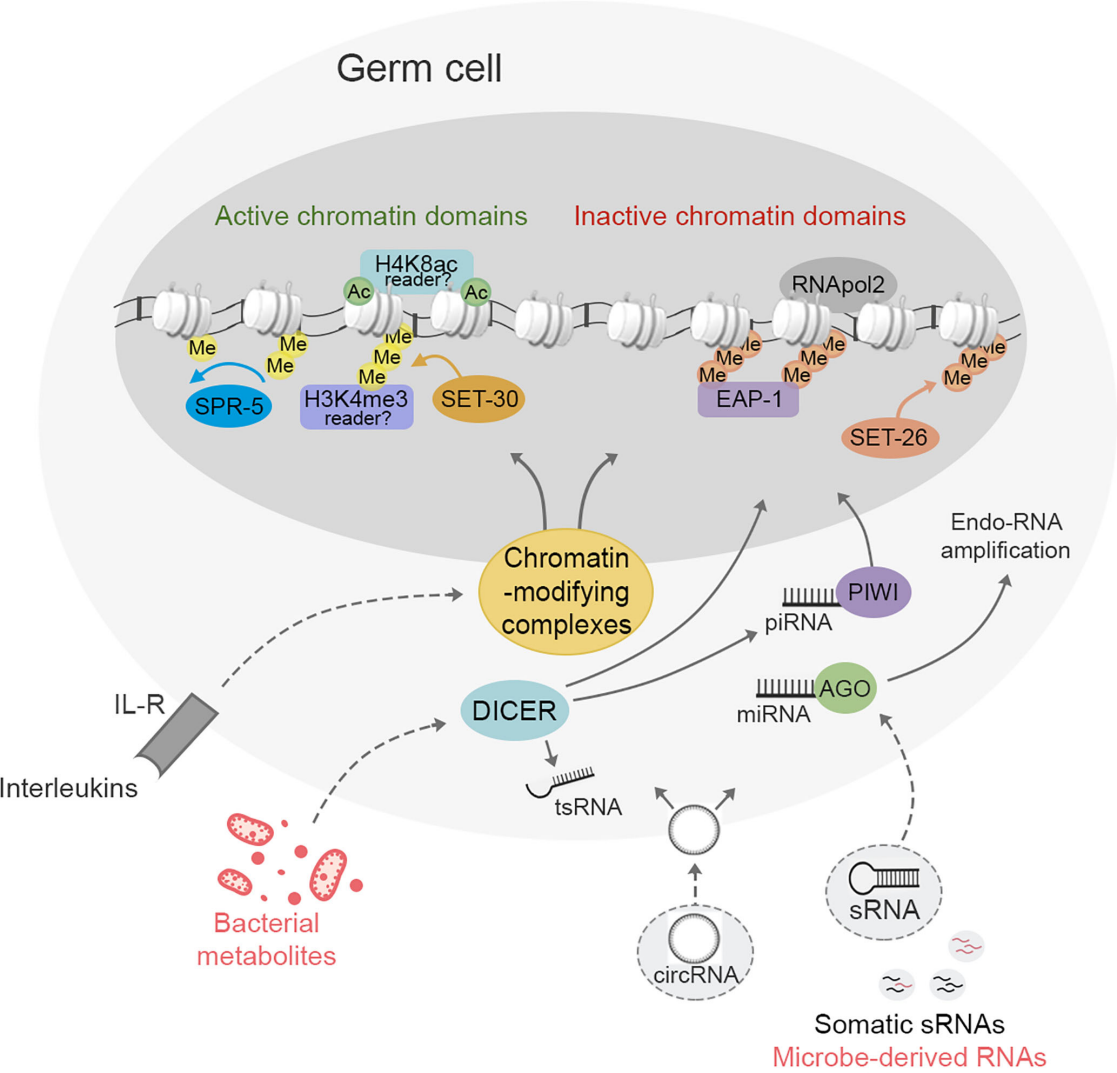
Environmental stimuli regulate epigenetic information in germ cells linked to transgenerational inheritance. In both vertebrates and invertebrates epigenetic changes in the germline lead to intergenerational or transgenerational inheritance. In *C. elegans*, these changes involve chromatin remodeling through activation of the H3K4 tri-methyltransferase SET-30, H3K4 demethylase SPR-5, H3K9 tri-methyltransferase SET-26 and the reader of H3K9me3, EAP-1. The remaining chromatin remodeling mechanisms and the mammalian orthologous involved in the establishment of the active and inactive chromatin domains inherited to the offspring are still poorly known. sRNAs and their processing machinery are central players in germ cells to inherit phenotypical traits across generations. piRNAs and PIWI proteins, miRNAs and AGO proteins, circRNAs and tsRNAs present in germ cells mediate transgenerational traits. sRNAs exert their effects via chromatin remodeling or through RNA amplification. The sRNA endonuclease DICER mediates the metabolism and amplification of sRNAs and the cleavage of tRNAs to produce tsRNAs. These RNAs are also epigenetic regulators, participate into RNA interference (RNAi) pathway, and directly inherits phenotypical traits across generations. Both chromatin remodeling processes and RNA pathways in germ cells are modulated by acquisition of somatic sRNAs during germ cell differentiation. It is also possible that bacteria-derived RNAs, bacterial metabolites and immune signals may also regulate transgenerational epigenetic marks in germ cells.

Adaptation to changing environments is essential for any living organism to survive and reproduce. Sensing environmental cues produces a memory that is inherited across several generations, granting short and long-term adaptive responses in all organisms. Inheritance of parental experiences enable behavioral responses in the offspring that can lead to faster recognition of pathogenic and non-pathogenic stimuli, even though individuals may have never been exposed before. Bacteria create a holobiont system with the host that regulates metabolism, immune responses and behavioral outcomes that influence transgenerationally-inherited strategies to survive pathogenesis. Bacteria and gut microbiota regulate different epigenetic mechanisms in both somatic and germ cells in the host modulating the genetic and epigenetic memory that is inherited across generations. To this end, bacteria modulate the gut-brain-germ cell axis to orchestrate and stimulate different pathways in which sRNAs carry most of the transmissible information. Different sRNAs are delivered to germ cells inducing chromatin changes that create particular genomic accessibility regions, which in turn regulate the gene expression program in the offspring (**Figure 1**). sRNAs are also amplified in germ cells by a RNAi machinery, allowing the transmission of the information throughout several generations. Although the mechanisms by which transgenerational changes are produced in germ cells are still poorly understood, histone modifications as well as endogenous and somatic sRNAs are involved in creating a transmissible memory in animals. Gut bacteria can also directly influence germ cells through bacteria-produced sRNAs and metabolites. Likewise, microbial-induced cytokines could induce epigenetic changes in germ cells that impact the phenotypical traits inherited by the host. Thus, gut bacteria influence the creation of an epigenetic memory in germ cells through indirect mechanisms *via* the gut-brain axis and sRNAs, but may also directly stimulate gametes through bacteria- derived sRNAs and metabolites.

The role of bacteria in the inheritance of behavior and memory for an organism is of great significance in understanding how phenotypic plasticity, adaptability and transgenerational inheritance occur in the species. Nevertheless, the precise mechanisms by which bacteria influence neuronal responses and reprogram the germline epigenome need to be unraveled. Understanding the mechanisms will allow us to better understand how bacteria coordinate the diversity of inherited behavioral outputs in the organisms and their adaptive responses.

## Concluding Remarks

**Figure 2:**
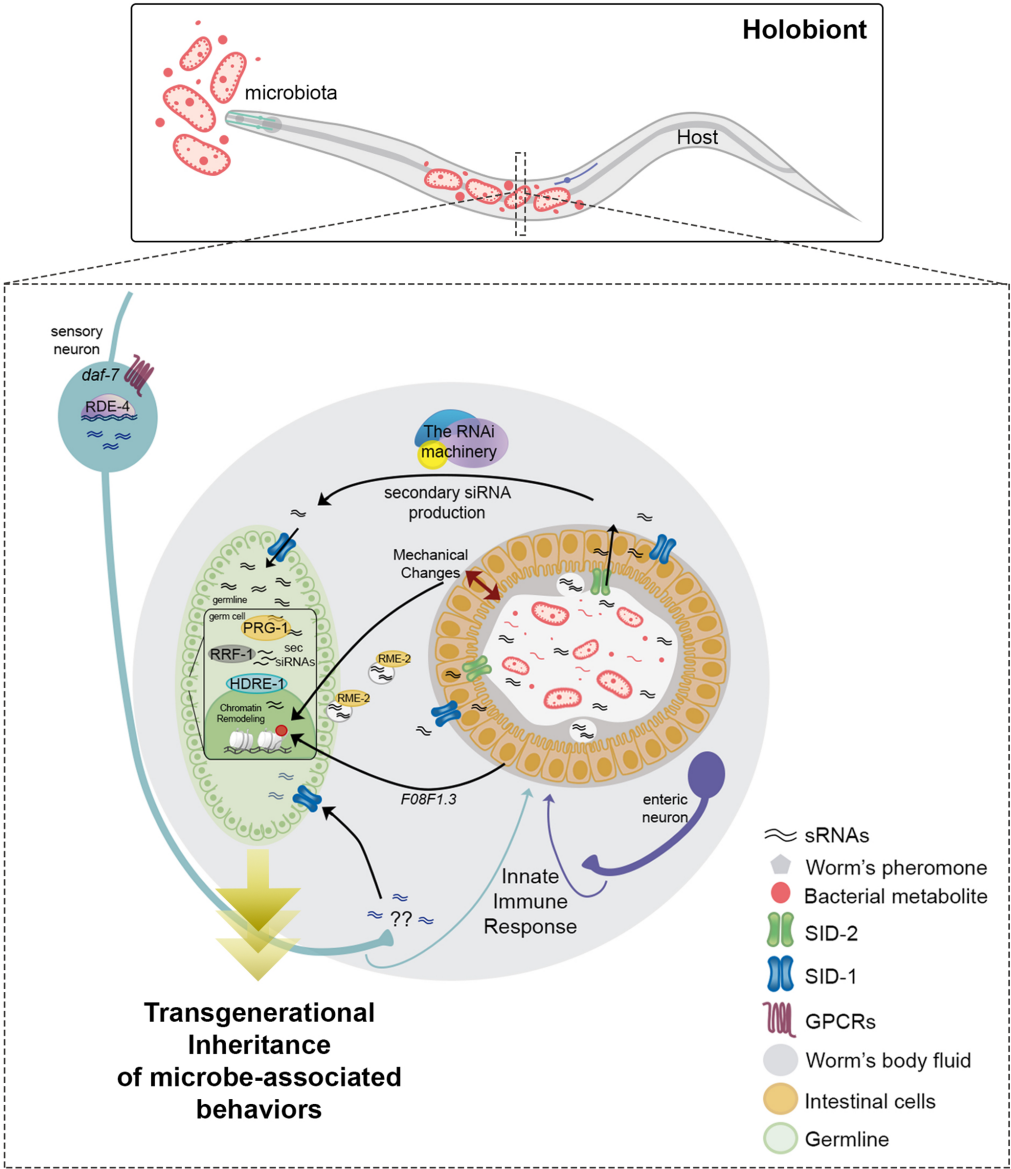
Small RNAs regulate transgenerational inheritance of behaviors triggered by previous microbial experience in *C. elegans*. Neuron to-gut to-germline signaling is essential to induce the transmission of the memory of previous microbial encounters. Sensory neurons perceive bacterial metabolites through GPCRs, modulating the DAF-7/TFG-β signaling pathway. Neuronal RDE-4 is necessary for sRNA production involved in transgenerational control of behavior. Neuronal sRNAs (blue) are transferred into germ cells through the dsRNA transporter SID-1 to induce transgenerational silencing. It is still unknown how these neuronally-derived sRNAs are secreted into the worms’ body. Sensory neurons (light blue) and neurons adjacent to the intestine (enteric; purple) control intestinal immunity against pathogens. Whether intestinal responses triggered by bacteria drive sRNAs changes is not known. Bacterial (pink) and host (black) sRNAs are transported from the lumen to the intestine through SID-2 and systemically through SID-1. RME-2 mediates the endocytosis of sRNAs from the worms’ cavity to oocytes. The RNAi machinery amplifies sRNAs causing transgenerational silencing. In the germline, the Piwi protein PRG-1, the RNA helicase RRF-1, and the nuclear Argonaute HDRE-1 are essential for driving transgenerational changes by epigenetic modulation. The transmission of epigenetic information between intestine and germ cells is mediated by the transcriptional regulation of F08F1.3. Mechanical changes in the intestine triggered by bacterial colonization may induce intergenerational histone acetylation in the germline.

The dynamic relationship between host and microbe forms a higher-order organism known as the *holobiont* (150, 151). The continuous interaction between them influences each other at metabolic, physiological, and at genetic levels (**Figure 2**).

As mentioned before, we hypothesized that for memories to be transmitted multigenerationally a quantitative threshold must be reached. Once the threshold is exceeded, the information must be “stamped” on the germ cells in order to be heritable. Epigenetics emerged as the main mechanisms from where information about environmental changes (e.g. toxicants presence, high fat diet, osmotic and thermal stress, caloric restriction, among others) is transmitted from parents to subsequent generations (152, 153). In *C. elegans* small RNAs produced by the host (4, 7, 36, 83, 84, 154–156) and bacteria (85, 86, 157) are primary effectors for driving environmentally induced heritable behaviors. However, how environmental changes are sensed and then transmitted to the progeny breaching the germline to soma barrier is still not fully understood.

In summary, in nematodes, priming or sensitivity of the progeny to a specific bacteria or bacterial byproduct is shaped by the previous generations experiences in a mechanism first decoded by sensory neurons, integrated by other neurons and intestinal cells, and then transmitted to the germ cells before fertilization. Additional studies are required to determine how bacterial host composition and microbe-derived byproducts are sensed by the neurons, integrated and precisely transmitted to the germline. However, neuronally produced small RNAs are essential to induce transgenerational gene silencing (83) and transgenerational behavioral memories (84) thus suggesting that the molecular signal accumulating over time is likely an RNA molecule. Future investigation will shed light on how the length and the virulence of the pathogen modulate the abundance of derived host and bacterial RNA molecules and the diversity of observed (and yet still undiscovered) inherited behavioral outputs in *C. elegans* (5, 8). However, in the contrary, transgenerational epigenetic inheritances in mammalian systems are only beginning to be unraveled. Recent work led by scientists in Australia demonstrates that the progeny of mouse infected with the parasitic protozoan *Toxoplasma gondii* exhibited abnormalities in anxiety, working memory, object recognition, sociability and mating behaviors. Surprisingly, the behavioral abnormalities displayed by the F1 and F2 were correlated with alterations in the RNA profile of previously infected F0 male gametes (158), and surprisingly, RNA extracted from infected sperm recapitulates the behavioral effects of the paternal infection. Thus, and as the French biologist Jacques Monod once said “What is true for *E. coli* is true for the elephant”, what is true for the worm it is also true for mouse and humans: we are just beginning to understand the importance of RNAs as key molecules in the evolution of the organisms as *holobionts*, a network of continuous dynamic interactions between and across the diverse biological levels of complexity that forms it.

## Author Contributions

All authors contributed equally to the writing, generation of figures, discussion and editing of the manuscript. All authors contributed to the article and approved the submitted version.

## Funding

Millennium Scientific Initiative ICM-ANID ICN09-022, CINV, Proyecto Apoyo Redes Formación de Centros (REDES180138), and CYTED grant P918PTE3 to AC; Fondecyt 11181329 to MM. MFP is funded by a doctoral fellowship (21161437) from the National Agency for Research and Development (ANID) and from ICM-ANID IC09-022.

## Conflict of Interest

The authors declare that the research was conducted in the absence of any commercial or financial relationships that could be construed as a potential conflict of interest.
